# Valsartan independent of AT_1_ receptor inhibits tissue factor, TLR-2 and-4 expression by regulation of Egr-1 through activation of AMPK in diabetic conditions

**DOI:** 10.1111/jcmm.12354

**Published:** 2014-08-11

**Authors:** Yu Mi Ha, Eun Jung Park, Young Jin Kang, Sang Won Park, Hye Jung Kim, Ki Churl Chang

**Affiliations:** a Department of Pharmacology, College of Medicine, Yeungnam UniversityDaegu, Korea; b Department of Pharmacology, School of Medicine, Institute of Health Sciences, Gyeongsang National UniversityJinju, Korea; c Present address: Department of Pharmacology, College of Medicine, Dong-A UniversityBusan, Korea

**Keywords:** valsartan, AMPK, Egr-1, diabetes, atherothrombosis

## Abstract

Patients suffering from diabetes mellitus (DM) are at a severe risk of atherothrombosis. Early growth response (Egr)-1 is well characterized as a central mediator in vascular pathophysiology. We tested whether valsartan independent of Ang II type 1 receptor (AT_1_R) can reduce tissue factor (TF) and toll-like receptor (TLR)-2 and-4 by regulating Egr-1 in THP-1 cells and aorta in streptozotocin-induced diabetic mice. High glucose (HG, 15 mM) increased expressions of Egr-1, TF, TLR-2 and-4 which were significantly reduced by valsartan. HG increased Egr-1 expression by activation of PKC and ERK1/2 in THP-1 cells. Valsartan increased AMPK phosphorylation in a concentration and time-dependent manner *via* activation of LKB1. Valsartan inhibited Egr-1 without activation of PKC or ERK1/2. The reduced expression of Egr-1 by valsartan was reversed by either silencing Egr-1, or compound C, or DN-AMPK-transfected cells. Valsartan inhibited binding of NF-κB and Egr-1 to TF promoter in HG condition. Furthermore, valsartan reduced inflammatory cytokine (TNF-α, IL-6 and IL-1β) production and NF-κB activity in HG-activated THP-1 cells. Interestingly, these effects of valsartan were not affected by either silencing AT_1_R in THP-1 cells or CHO cells, which were devoid of AT_1_R. Importantly, administration of valsartan (20 mg/kg, i.p) for 8 weeks significantly reduced plasma TF activity, expression of Egr-1, TLR-2,-4 and TF in thoracic aorta and improved glucose tolerance of streptozotocin-induced diabetic mice. Taken together, we concluded that valsartan may reduce atherothrombosis in diabetic conditions through AMPK/Egr-1 regulation.

## Introduction

Tissue factor (TF) is an important protein, not only as the key trigger of the coagulation cascade, but also as a mediator in the pathogenesis of cardiovascular disorders. Indeed, TF antigen is elevated in diabetic patients with microvascular complications, and TF activity is reduced in diabetic patients with improved glycaemic control 1,2. The magnitude of the thrombotic processes triggered by plaque disruption is dependent upon the levels of TF 3. TF is the principal initiator and propagator of thrombus formation in the extrinsic coagulation system. Atherosclerotic plaques contain significant levels of TF mRNA and antigens that are localized within macrophages and smooth muscle cells in the plaques 4. TF in these plaques can bind to factor VIIa, and the TF:VIIa complex can activate factor IX and factor X 5. Finally, factor Xa catalyses formation of thrombin, which causes thrombus formation in ruptured plaques.

Hyperglycaemia is a major contributor to the cardiovascular diseases associated with diabetes. Patients suffering from diabetes mellitus (DM) are at a severe risk of atherothrombosis, largely because of an increase in the levels of several pro-coagulation factors (*e.g*. fibrinogen, factor VII and von Willebrand factor) and a decrease in the levels of several anticoagulation factors (*e.g*. antithrombin III and protein C) 6. In fact, atherothrombosis accounts for the majority of deaths in patients with DM 7. These patients are also at high risk for peripheral vascular disease, cerebrovascular disease and stroke 8. Recent reports have suggested that inflammation and innate immunity can be considered as important regulators of pathogenesis of DM. Increased levels of toll-like receptor (TLR)-2, TLR-4 expression, activity and signalling in monocytes have been reported in type 1 diabetic patient 9. In addition, involvement of TLR-9 and early growth response factor 1 (Egr-1) has been reported to induce foam cells and TF expression in RAW 264.7 cells 10. By acting as a master transcription factor, Egr-1, a zinc finger nuclear protein, regulates a set of genes implicated in the pathogenesis of atherosclerosis, with subsequent thrombosis and restenosis 11,12. A close link between effects of Ang II receptor blockers (ARBs) and AMP-activated protein kinase (AMPK) activity has been reported on glucose metabolism in an animal experiment 13. However, it is not clear that improved glucose homoeostasis after Ang II type 1 receptor (AT_1_R) blockade is also related to an AT_1_-independent, but to Egr-1-dependent mechanism. Thus, the aim of the current study was to determine whether valsartan inhibits TLR-2,-4 and TF in HG-activated THP-1 cells and vascular tissues in streptozotocin (STZ)-induced type 1 DM mice by suppression of Egr-1 expression *via* AMPK activation independent of AT_1_R.

## Methods and methods

### Materials

RPMI 1640 medium and fetal bovine serum (FBS) and antibiotics (penicillin and streptomycin) were purchased from Gibco BRL (Rockville, MD, USA). Anti-AMPK and anti-PKC antibody were purchased from Cell Signaling Technology (Danvers, MA, USA). Anti-Egr-1 antibody, anti-TLR-2, anti-TLR-4, horseradish peroxidase (HRP) labelled goat anti-rabbit IgG, donkey anti-goat IgG and anti-ERK1/2 antibodies were purchased from Santa Cruz Biotechnology (Santa Cruz, CA, USA). Anti-β-actin was purchased from Sigma-Aldrich (St. Louis, MO, USA). PD98059 and Gö6976 were purchased from Calbiochem (San Diego, CA, USA). Enhanced chemiluminescence (ECL) and Western blotting detection reagent were purchased from Amersham (Buckinghamshire, UK). Phorbol 12-myristate 13-acetate, 5-aminoimidazole-4-carboxamide riboside (AICAR) and compound C were purchased from Sigma-Aldrich. Valsartan was kindly supplied from Novartis Pharma AG (Bazel, Switzerland).

### Cell culture

A human monocytic cell line, THP-1 and a Chinese hamster ovary cell line, CHO cells, were obtained from the American Type Culture Collection (ATCC, Rockville, MD, USA). The cells were grown in Roswell Park Memorial Institute 1640 medium (RPMI 1640), DMEM and DMEM-Ham's F-12K medium, respectively, supplemented with 100 U/ml penicillin, 100 μg/ml streptomycin and 10% heat-inactivated FBS.

### Cell stimulation

THP-1 cells were plated at a density of 1 × 10^6^ cells per ml in a 60-mm dish. To induce macrophage phenotype differentiation, 50 ng/ml phorbol 12-myristate 13-acetate was added to the culture. After 24 hrs, non-adherent cells and PMA were washed off three times with PBS, and the adherent macrophages were incubated in RPMI 1640 medium and DMEM supplemented with penicillin and 10% FBS for a further 2–5 days.

### Western blot analysis

Total protein was acquired using lysis buffer containing 0.5% SDS, 1% Nonidet P-40, 1% sodium deoxycholate, 150 mM NaCl, 50 mM Tris–Cl (pH 7.5) and protease inhibitors. The protein concentration of each sample was determined using a BCA protein assay kit (Pierce, Rockford, IL, USA). Forty microgram aliquots of the protein were electrophoresed on 10% polyacrylamide gels for detection of AMPK or Egr-1, TLR-2 and-4, ERK1/2, and β-actin. The electrophoresed proteins were transferred to polyvinylidene difluoride (PVDF) membranes by semidry electrophoretic transfer at 15 V for 60–75 min. The PVDF membranes were blocked overnight at 4°C in 5% bovine serum albumin (BSA). The cells were incubated with primary antibodies diluted 1:500 in Tris-buffered saline/Tween 20 (TBST) containing 5% BSA for 2 hrs, followed by incubation with the secondary antibody at room temperature for 1 hr. Anti-rabbit IgG and anti-goat IgG were used as the secondary antibody (1:5000 dilution in TBST containing 1% BSA). Signals were detected by ECL (Amersham, Piscataway, NJ, USA). Scanning densitometry was performed with an Image Master® VDS (Pharmacia Biotech Inc., San Francisco, CA, USA).

### Measurement of secreted TNF-α, IL-6 and IL-1β in culture cells by ELISA

Levels of TNF-α, IL-6 and IL-1β in the conditioned medium were determined using TNF-α, IL-6 and IL-1β enzyme-linked immunosorbent assay kits, respectively (R&D Systems, Minneapolis, MN, USA) according to the manufacturer's instruction. The cells were pre-treated with or without valsartan, followed by HG stimulation for 4 or 24 hrs.

### Transient transfection assay

THP-1 cells were seeded into six-well tissue culture plates at 1 × 10^6^ cells per well 18–24 hrs prior to transfection. After incubation for 4 hrs, the medium was replaced with fresh medium. Following incubation for 24 hrs, cells were then incubated for different periods (1, 8 and 48 hrs) in medium containing HG or valsartan.

### Small interfering RNA technique

Small interfering RNAs (siRNAs) against human Egr-1 and scramble siRNA were purchased from Santa Cruz Biotechnology and used according to the manufacturer's protocol using transfection reagent SuperFect® from Qiagen (Hilden, Germany). The cells were incubated with 30 nM Egr-1 siRNA or 20 nM AT_1_R siRNA for 24 hrs in serum, antibiotics and FBS, and cells were washed and pre-treated with or without valsartan, followed by HG stimulation.

### TF activity assay

Cells were plated to a density of 1×10^4^ cells per well and TF activity in THP-1 and EA.hy926 cells was measured using an enzyme-linked chromogenic assay kit for the TF activity assay kit (American Diagnostica Inc., Stamford, CT, USA) according to the manufacturer's protocol. The cells were pre-treated with or without valsartan, followed by HG stimulation for 24 hrs.

### Luciferase assay

After experimental treatments, cells were washed twice with cold PBS, lysed in a lysis buffer provided in the dual luciferase kit (Promega, Madison, WI, USA) and assayed for luciferase activity using a TD-20/20 luminometer (Turner Designs, Sunnyvlae, CA, USA) according to the manufacturer's protocol. All transfections were performed in triplicate. The β-galactosidase assay was performed according to the supplier's instructions (Promega β-galactosidase Enzyme Assay System) for normalizing luciferase activity. The data are presented as a ratio between Firefly and Renilla luciferase activity.

### Chromatin immunoprecipitation (ChIP) assay

This assay was performed with the ChIP assay kit from Cell Signaling Technology (Beverly, MA, USA) according to the manufacturer's instructions. THP-1 cells were cultured with drug at the indicated time and later stimulated with HG, followed by fixation in 1% formaldehyde for 10 min. at room temperature. Cross-linking was stopped by addition of glycine. DNA was digested using Micrococcal Nuclease to a length of ∼150–900 bp. Chromatin was then incubated with 10 μg Egr-1 and NF-κB antibody at 4°C (Santa Cruz Biotechnology). Immune complexes were precipitated, washed, and eluted as recommended. DNA protein cross-links were reversed by heating at 65°C for 2 hrs. Of each sample 10 μl were used as a template for PCR amplification. TF oligonucleotide sequences for PCR primers were forward-5′-GCCCTCCCTTTCCTGCCATAGA-3′ and reverse-5′-CCTCCCGGTAGGAAACTCCG-3′. This primer set encompasses the TF promoter segment from nucleotide +121 to −227. PCR was performed at 94°C for 30 sec., 60°C for 30 sec., 72°C for 30 sec., and ending with 5 min. at 72°C. The identities of TF promoter were confirmed. Each separate experiment was repeated at least twice.

### Angiotensin II concentration measurement

Angiotensin II levels were measured using antibody ELISA following the protocol given by commercial antibody pairs, recombinant standards and a streptavidin-HRP detection system (ENZO Life Sciences, Ann Arbor, MI, USA). The cells were treated with glucose (0, 5, 15, 25 and 35 mM), following HG stimulation of 24 hrs. In brief, a 96-well microplate was pre-coated with 50 μl of the samples to the bottom of the appropriate wells. Recombinant angiotensin II was used to set up the standard curve. And then, all reagents were brought to room temperature and prepared according to the manufacturer's directions. The O.D at 450 nm was measured using microplate reader (M960; Metertech, Taipei, Taiwan). Intra-and inter-assay precision CVs are ≤10.0%.

### Animal experiment

Diabetes was induced by intraperitoneal injection (i.p.) of 40 mg/kg STZ dissolved in citrate buffer with pH 4.5 in the morning between 9:00 and 10:00 hrs for five consecutive days into 8-to 10-week-old male C57BL/6 mice, which were obtained from JungAng Inc. (Osan, Korea). The mice were maintained with free access to water and chow throughout the study period and were treated in accordance with the guidelines for animal experimentation of our institute. Mice were maintained in accordance with the Guide for the Care and Use of Laboratory Animals (NIH publication 85-23, revised 1996) and were treated ethically. The protocol was approved in advance by the Animal Research Committee of Gyeongsang National University. Forty four mice were divided into the following five groups: (1) vehicle-treated mice (*n* = 7), (2) STZ-induced diabetic mice (*n* = 10), (3) valsartan (10 mg/kg) + STZ-induced diabetic mice (*n* =* *10), (4) valsartan (20 mg/kg) + STZ-induced diabetic mice (*n* = 10) and (5) valsartan (20 mg/kg)-treated mice (*n* = 7). Valsartan was dissolved in 0.1% potassium hydroxide and administered i.p for 8 weeks at dose of 10 mg/kg and 20 mg/kg. After 8 weeks of treatment according to experimental procedure, each group of animals was subjected to measure plasma glucose, plasma insulin level and plasma TF activity. After checking these parameters, thoracic aorta was excised and performed Western blot analysis for Egr-1, TLR-4 and TF expression.

### Glucose tolerance test

After fasting overnight, each group of mice was injected with 2 g/kg bodyweight of glucose (i.p). Blood glucose levels were then measured at indicated times (0, 30, 60, 120 and 180 min.) using a portable glucose meter (LifeScan, Milpitas, CA, USA) after tail snipping. Simultaneously, blood samples were collected for examination of insulin concentration.

### Blood insulin assays

ELISA was performed for determination of plasma insulin levels following the protocol given by the manufacturer (Crystal Chem Inc., Downers Grove, IL, USA). In brief, 95 μl of sample diluent and 5 μl samples were added to a 96-well microplate pre-coated with polyclonal antibody specific for mouse insulin. Recombinant mouse insulin was used to set up the standard curve. After incubation for 2 hrs at 4°C, the wells were washed and polyclonal antimouse insulin antibodies conjugated to HRP were added. Incubation was continued for 30 min. at room temperature, plates were washed; substrate solution was added to each well, followed by incubation for 40 min. The enzyme reaction yielded a blue product that turned yellow when the stop solution was added. The O.D was measured at 450 nm (correction wavelength set at 630 nm) using a microplate reader (M960; Metertech).

### Statistical analysis

Treatment groups were compared using one-way anova and the Newman–Keuls test was used to locate significant differences identified in the anova. The data represent as mean ± SEM. *P* < 0.05 or *P* < 0.01 was accepted as significant.

## Results

### Valsartan increases phosphorylation of AMPK through LKB-1 in THP-1 cells

**Figure 1:**
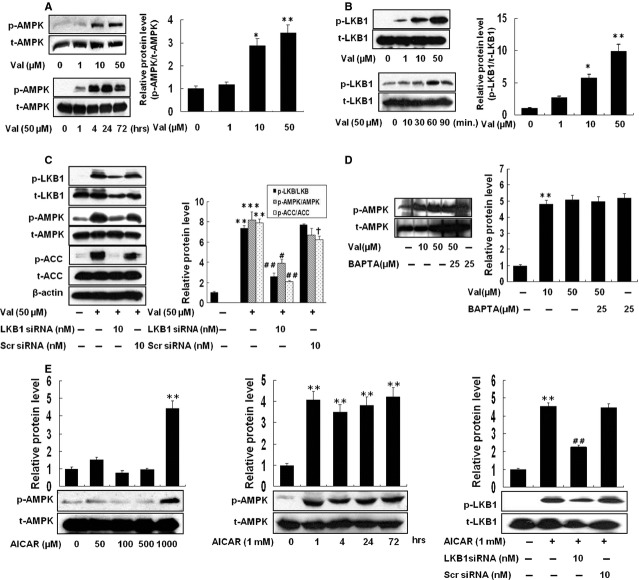
Valsartan activates AMPK *via*LKB1 signal but not CaMKK. The levels of phosphorylation of AMPK were investigated in THP-1 cells by incubating with different concentrations and different time periods of valsartan. After finishing incubation, cells were harvested and proteins were quantified. Using corresponding antibodies (p-AMPK, AMPK, p-LKB1 and LKB1), Western blot was performed (**A** and **B**). To confirm that valsartan activates AMPK through LKB1, cells were transfected with LKB1 siRNA or scrambled siRNA, as described in Methods and incubated with valsartan for 24 hrs. The proteins were quantified and Western blot analysis was performed with corresponding antibodies (P-AMPK, AMPK, p-LKB1, LKB1, p-ACC and ACC). The relative intensity of each protein is depicted as bar graphs on the right side (**C**). The level of phosphorylation of AMPK was determined by valsartan in the presence or absence of BAPTA (**D**). For positive control, the levels of p-AMPK and p-LKB1 were investigated using AICAR with the same methods described above (**E**). The blots shown are representative ones with similar results of three independent experiments. Results are expressed as the means ± SEM of three independent experiments. *, ** indicate *P* < 0.05, *P* < 0.01 compared to control, respectively. #, ## indicate *P* < 0.05, *P* < 0.01 compared to valsartan, respectively. †*P* < 0.05 compared to valsartan.

As shown in Figure1A, valsartan induced an increase in phosphorylation of AMPK in a time-and concentration-dependent manner. Two upstream kinases, including LKB1 and calcium/calmodulin-dependent kinase kinase (CaMKK)-β, have been implicated in phosphorylation of AMPK α subunit at Thr^172^ 14. Valsartan induced an increase in phosphorylation of LKB1 in a concentration and time-dependent manner (Fig.1B). Valsartan (50 μM) induced a significant increase in p-AMPK and p-LKB1, which were significantly inhibited by silencing of LKB1, but not by scramble siRNA-transfected cells (Fig.1C). On the other hand, the level of expression of p-AMPK by valsartan was not affected when BAPTA, a Ca^2+^ chelator, was introduced (Fig.1D). AICAR also increased p-AMPK in a concentration and time-dependent manner and silencing LKB1 significantly reduced the expression of p-LKB1 (Fig.1E).

### High glucose increases Egr-1 expression *via* PKC and ERK1/2 MAPK activation

**Figure 2:**
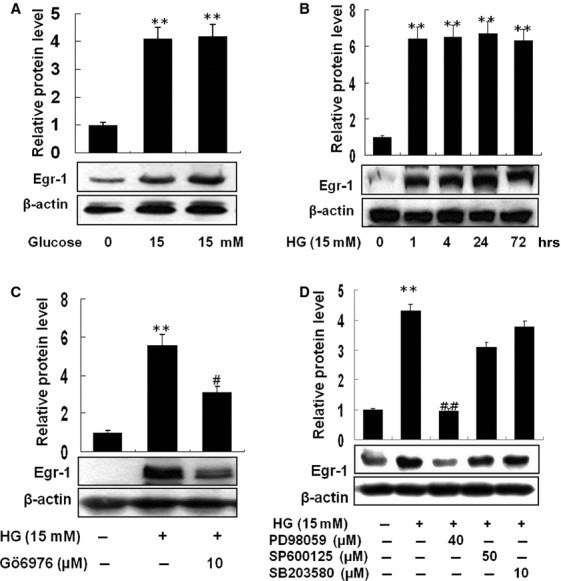
High glucose increases Egr-1 protein expression *via*PKC and ERK1/2 pathways. The level of Egr-1 expression was investigated by treatment of THP-1 cells with high glucose (15 mM). After incubation for 1 hr, proteins were quantified and equal amounts of proteins were loaded and Western blot analysis was performed with anti-Egr-1 antibody (**A**). Cells were treated with high glucose (15 mM) and incubated for the indicated time periods. Western blot analysis using anti-Egr-1 antibody showed that cells treated with high glucose (HG) began to show an increase in Egr-1 at 1 hr and was sustained until 72 hrs (**B**). Increased expression of Egr-1 by HG was diminished by Gö6976 (10 μM), a PKC inhibitor (**C**). Among MAPK inhibitors, only PD98059 (40 μM) but not SP60015 (50 μM) and SB203580 (10 μM) significantly reduced HG-activated Egr-1 expression (**D**). Results are expressed as the means ± SEM of three independent experiments. ** indicate *P* < 0.01 compared to control. #, ## indicate *P* < 0.05, *P* < 0.01 compared to HG, respectively.

By acting as a master transcription factor, Egr-1, a zinc finger nuclear protein, regulates a set of genes implicated in the pathogenesis of atherosclerosis, with subsequent thrombosis and restenosis 15. As shown in Figure2A, stimulation with HG (15 mM) induced a significant increase in expression of Egr-1 transcription factor. In particular, the increase of Egr-1 expression by HG started as early as 1 hr and lasted until 72 hrs (Fig.2B). Protein kinase C and MAPK are known to increase Egr-1 expression 15,16. Thus, we asked which signal(s) are involved in HG-induced up-regulation of Egr-1 expression. Pre-treatment with Gö6976, a PKC inhibitor, resulted in significantly reduced expression of Egr-1 (Fig.2C). Next, when checked with different MAPK inhibitors, only PD98059, an ERK1/2 MAPK inhibitor, sensitively inhibited Egr-1 expression by HG (Fig.2D).

### Valsartan inhibits Egr-1 expression *via* AMPK activation

**Figure 3:**
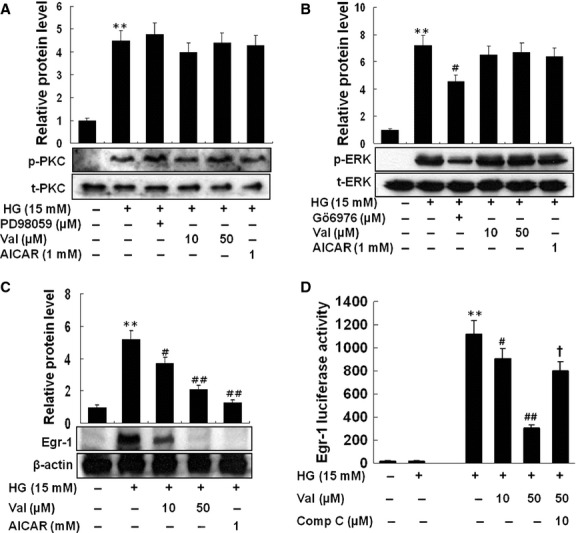
Valsartan inhibits HG-induced Egr-1 expression *via* the AMPK-dependent pathway. To determine the relation between PKC and ERK1/2 in HG-induced Egr-1 expression, effect of PD98059 (40 μM) or Gö6976 (10 μM) on expression of PKC and p-ERK was investigated. HG induced a significant increase in p-PKC; however, neither PD98049, nor valsartan, nor AICAR affected HG-induced PKC expression (**A**). In contrast, increased p-ERK by HG was significantly reduced by Gö6976, a PKC inhibitor, indicating that PKC is located upstream of ERK (**B**). The level of Egr-1 expression was investigated in HG-activated cells treated with different concentrations of valsartan (10, 50 μM) or AICAR (1 mM). After incubation for 1 hr, proteins were quantified and equal amounts of proteins were loaded; Western blot analysis was performed with anti-Egr-1 antibody (**C**). Egr-1 luciferase activity was measured in cells transiently transfected with Egr-1 luciferase, as described in Methods. Different treatment (valsartan 10, 50 μM, and valsartan 50 μM + compound C 10 μM) was performed 30 min. before addition of high glucose (HG, 15 mM), and incubated for 1 hr (**D**). Results are expressed as the means ± SEM of three independent experiments. ** indicate *P* < 0.01 compared to control. #, ## indicate *P* < 0.05, *P* < 0.01 compared to HG, respectively. †*P* < 0.05 compared to valsartan.

As PKC/ERK1/2 signals are important for induction of Egr-1 expression by HG in THP-1 cells, we asked whether valsartan affects activity of PKC and/or ERK1/2 in HG-activated THP-1 cells. As shown in Figure3A, unexpectedly, HG-induced phosphorylation of PKC was not affected by either valsartan or AICAR. Likewise, either valsartan or AICAR did not, but Gö6976 (PKC inhibitor) did diminish the expression of p-ERK in HG-activated THP-1 cells (Fig.3B). It should be noted that although phosphorylation of PKC by HG was not affected by PD98059 (ERK inhibitor), but phosphorylation of ERK1/2 by HG was significantly reduced by Gö6976, indicating that ERK1/2 MAPK is located downstream of PKC in the regulation of Egr-1 (Fig.3A and B). Because valsartan significantly reduced HG-induced Egr-1 expression independent of PKC and ERK1/2 activity (Fig.3C), we speculated that AMPK signal may be responsible for this event. As expected, valsartan significantly reduced Egr-1 luciferase activity by HG, which was reversed by compound C (Fig.3D) confirming that activation of AMPK by valsartan reduces Egr-1 in HG-activated THP-1 cells.

### Valsartan inhibits NF-κB and Egr-1 binding to HG in the TF promoter

**Figure 4:**
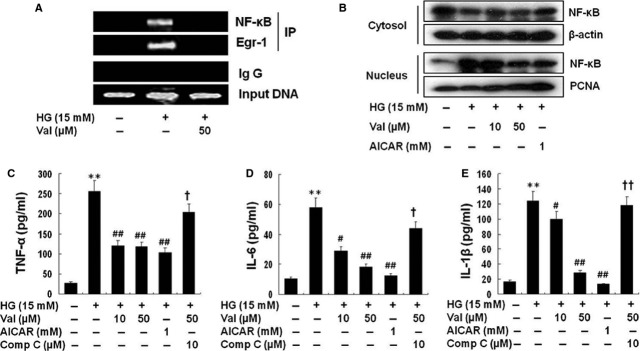
Valsartan inhibits NF-κB translocation and activity in HG-activated cells. Chromatin immunoprecipitation (ChIP) showed that NF-κB and Egr-1 were co-precipitated with HG-activated cells, in which valsartan treatment obligated binding of these transcription factors to the TF promoter (**A**). Cells were treated with different concentrations of valsartan (10, 50 μM) or AICAR (1 mM) 30 min. before application of high glucose (HG, 15 mM) and incubated for 1 hr. Then, cytosol and nuclear fraction were isolated as described in Methods. Each fraction was immunoblotted with anti-NF-κB (p65) antibody (**B**). Because valsartan inhibited NF-κB, production of pro-inflammatory cytokines (TNF-α, IL-6 and IL-1β) by HG was inhibited by valsartan was investigated as described in Methods. (**C, D, E**) It shows that valsartan significantly reduced production of pro-inflammatory cytokines in a compound c-sensitive manner. Results are expressed as the means ± SEM of three independent experiments. ** indicate *P* < 0.01 compared to control. #, ## *P* < 0.05, *P* < 0.01 compared to HG, respectively. †, †† *P* < 0.05, *P* < 0.01 compared to valsartan (50 μM) treatment, respectively.

NF-κB is an important regulator of inflammation and TF expression. Because we found that Egr-1 was a target molecule for the action of valsartan, it is of great interest to know whether valsartan inhibits binding of NF-κB and Egr-1 to TF promoter in HG condition. Therefore, ChIP was performed. NF-κB and Egr-1 were co-precipitated with HG-activated cells, in which valsartan treatment obligated binding of these transcription factors to the TF promoter (Fig.4A). As expected, valsartan and AICAR inhibited translocation of NF-κB from cytosol to nucleus by HG (Fig.4B). In addition, valsartan significantly reduced pro-inflammatory cytokine production such as TNF-α (Fig.4C), IL-6 (Fig.4D) and IL-1β (Fig.4E) by HG-activated THP-1 cells, which were significantly reversed by compound C. AICAR also showed the anti-inflammatory effect (Fig.4C–E).

### Valsartan inhibits HG-activated TF expression and activity by AMPK-dependent pathway

**Figure 5:**
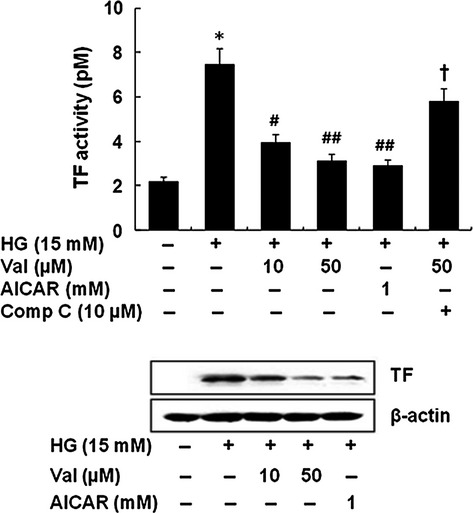
Valsartan inhibits TF expression and activity by an AMPK-dependent manner. Cells were treated with different concentrations of valsartan (10, 50 μM), AICAR (1 mM), and valsartan (50 μM) + compound C (10 μM) 30 min. before addition of high glucose (HG, 15 mM) and incubated for 24 hrs. Cell lysates were harvested and TF activity was measured using an enzyme-linked chromogenic assay kit. After incubation for 24 hrs, proteins were quantified and equal amounts of proteins were loaded; Western blot analysis was performed with anti-TF antibody. Results are expressed as the means ± SEM of three independent experiments. * indicates *P* < 0.05 compared to control. #, ## *P* < 0.05, *P* < 0.01 compared to HG. † indicates *P* < 0.05 compared to valasartan.

Figure5 shows that valsartan significantly reduced TF expression as well as activity that increased by HG in THP-1 cells, which was reversed by compound C. AICAR also significantly reduced TF activity and protein expression in HG-activated THP-1 cells.

### Valsartan reduces TF and TLR expression *via* Egr-1

**Figure 6:**
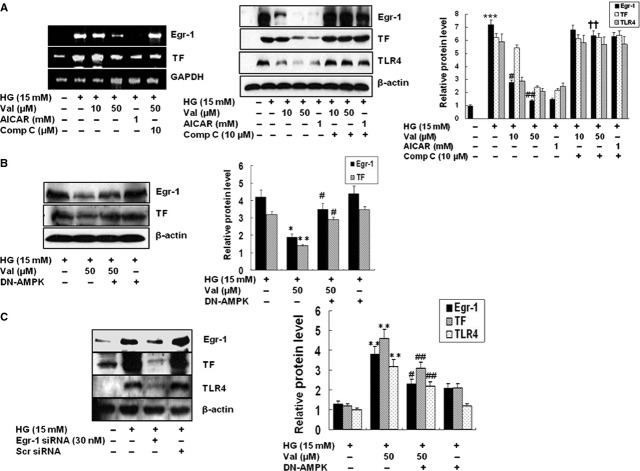
Valsartan inhibits TLR-4 and TF expression in an Egr-1-senstitive manner. Reporter cells were treated with different concentrations of valsartan (10, 50 μM) or AICAR (1 mM) 30 min. before application of high glucose (HG, 15 mM) and incubated for 1 hr for detection of Egr-1 and 24 hrs for detection of TLR-4. After mRNA and proteins were quantified, equal amounts were loaded for RT-PCR and Western blot analysis (**A**). The intensity of relative protein levels is depicted as bar graphs on the right side. To confirm involvement of Egr-1 in inhibition of TF, cells were transfected with DN-AMPK, which also shows AMPK dependency on inhibition of Egr-1 expression by valsartan (**B**). To further confirm the above results, cells were transfected with Egr-1 siRNA, and expression level of Egr-1, TLR-4 and TF was measured as described in Methods (**C**). The intensity of relative protein levels is depicted as bar graphs on the right side. Results are expressed as the means ± SEM of three independent experiments. *, ** indicate *P* < 0.05, *P* < 0.01 compared to control, respectively. #, ## indicate *P* < 0.05, *P* < 0.01 compared to HG, respectively. ††*P* < 0.01 compared to valsartan.

Figure6A shows that valsartan significantly reduced expressions of mRNA and protein of Egr-1, TF and TLR-4 by HG which were reversed by compound C. As expected, the effect of valsartan on expression of Egr-1 and TF was significantly reversed in DN-AMPK-transfected cells (Fig.6B). AICAR also diminished the expression of Egr-1 and TF at transcriptional (Fig.6A) as well as translational level (Fig.6B) which was significantly reversed by compound C, suggesting that AMPK activity controls the expression of Egr-1 and TF. Mechanisms of innate immunity and TLRs are implicated in inflammation and diabetes 17,18, therefore it is of interest to determine whether reduced expression of TLR and TF by valsartan is linked with Egr-1. As shown in Figure6C, HG-induced increase of TLR-4 and TF expression was significantly reduced by Egr-1 siRNA-transfected cells. In contrast, control siRNA-transfected cells were not affected. These results confirm that AMPK activity negatively regulates Egr-1 which influences expression of TF and TLR-4 in HG conditions.

### AT_1_R independent effect of valsartan

**Figure 7:**
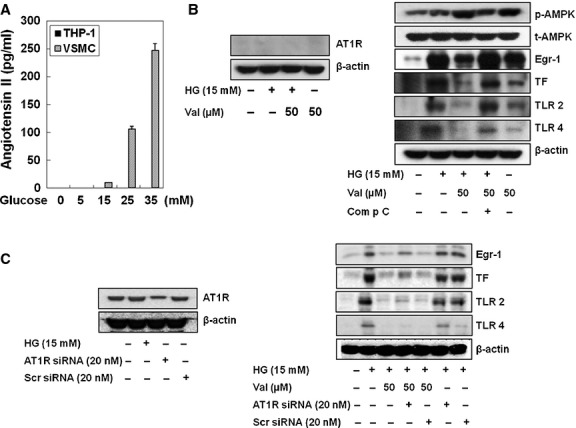
AMPK activation by valsartan is not associated with production of neither AT_1_R nor Ang II production by HG. To determine whether the reduction of Egr-1 expression by valsartan in HG-activated THP-1 cells is related to AT_1_R blocking action or HG-increased Ang II concentration in the media, concentration of Ang II produced by HG was measured. When vascular smooth muscle cells (VSMC) was used as positive control, glucose-induced concentration-dependent production of Ang II in VSMC, but not THP-1 cells (**A**). In addition, valsartan induced an increase in p-AMPK in CHO cells, which was reportedly devoid of AT_1_R, where valsartan induced a significant reduction of HG-induced Egr-1, TLR-2 and-4 and TF in a compound c-sensitive manner (**B**). To further confirm of this result, we exploited the siAT_1_R-RNA transfection technique (**C**). Increased expression of Egr-1, TF, TLR-2 and-4 by HG was significantly reduced by valsartan in both scramble siRNA-transfected cells and siAT_1_R-RNA-transfected cells. The blots shown are representative ones with similar results of three independent experiments.

The question remains that these effects of valsartan are reflection of AT_1_R blockade or concentration levels of Ang II in HG-conditioned media? To unveil this question, we measured Ang II production in THP-1 cells and CHO cells. For positive control, we used vascular smooth muscle cells (VSMC). Figure7A shows that the levels of Ang II were increased by HG concentration in a dose-dependent manner in VSMC; however, such an effect was not demonstrated in THP-1 cells (Fig.7A). To further confirm that the effect of the above mentioned valsartan is independent of AT_1_R, we used CHO cells, which were reported to be devoid of AT_1_R. As shown in Figure7B left panel, neither HG nor valsartan influenced AT_1_R expression in CHO cells. Figure7B right panel showed that even in CHO cells, valsartan reduced HG-induced expression of Egr-1, TF, TLR-2 and-4 *via* AMPK activation, as all of these effects were reversed by compound C. In addition, we found that valsartan was even able to reduce HG-induced expression of Egr-1, TF, TLR-2 and-4 in AT_1_R-siRNA-transfected THP-1 cells (Fig.7C). Transfection efficiency of AT_1_R-siRNA was shown in left panel of Figure7C.

### Valsartan significantly improves glucose tolerance and reduces expressions of TF, TLR-4 and Egr-1 in thoracic aorta of STZ-induced diabetic mice

**Figure 8:**
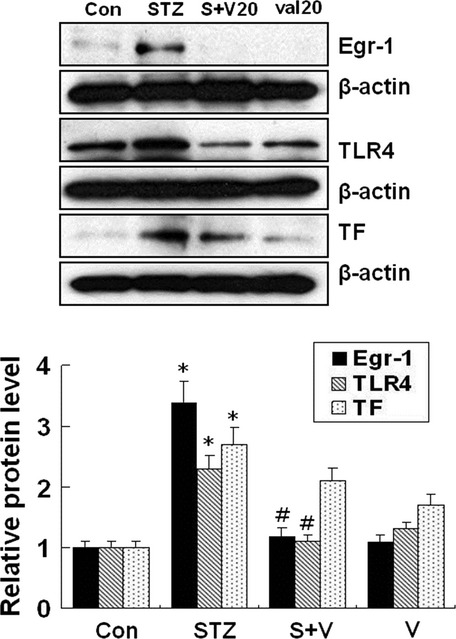
Effect of valsartan on Egr-1, TF, TLR-4 expression in aorta of streptozotocin-induced diabetic mice. Thoracic aorta was isolated from each group of animals as described in Methods (sham, streptozotocin, streptozotocin with valsartan and valsartan-treated group). Expression of Egr-1, TLR-4 and TF was determined by Western blot analysis. The blots shown are representative ones with similar results of three independent experiments. Results are expressed as the means ± SEM of each group of animals. * indicates *P* < 0.05 compared to control. # indicates *P* < 0.05 compared to STZ.

**Figure 9:**
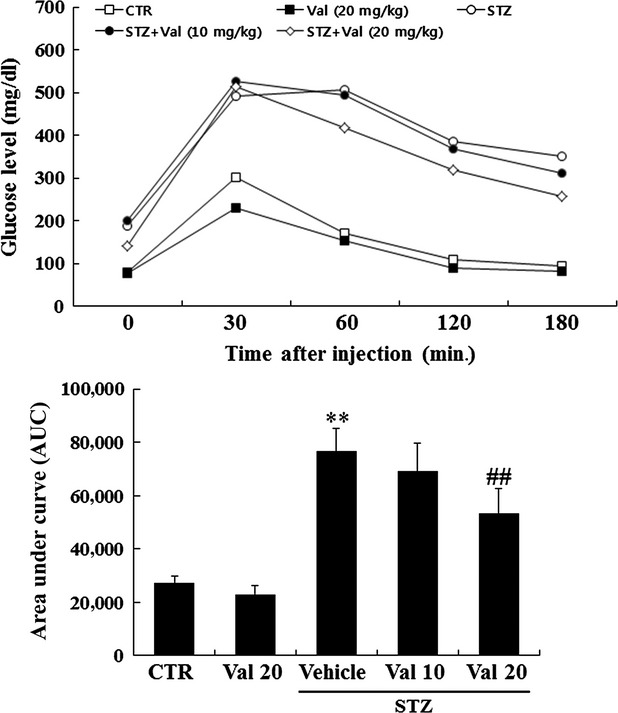
Effect of valsartan on intraperitoneally injected glucose tolerance test. The glucose tolerance test measures the clearance of an intraperitoneally injection with 2 g/kg body weight of glucose load from the body. Animals are fasted for ∼16 hrs, a solution of glucose is administered by intraperitoneal (i.p) injection, and blood glucose is measured at different time-points (0, 30, 60, 120 and 180 min.) during the following 3 hrs after tail snipping. Results are expressed as the means ± SEM of each group of animals. ** indicates *P* < 0.01 compared to control. ## indicates *P* < 0.01 compared to vehicle.

Table1 shows changes of bodyweight, blood glucose, blood insulin levels and TF activity after 8 weeks of animals (sham control, STZ, STZ+ valsartan (V) and V-treated mice). During 8 weeks, STZ treatment overtly increased plasma glucose and plasma TF activity which accompanied by weight loss and plasma insulin level. Although valsartan (10 mg/kg) tended to reduce the plasma glucose levels in type 1 diabetic mice, it is not statistically significant. However, treatment 20 mg/kg of valsartan significantly reduced the plasma glucose compared to STZ group (336.3 mg/ml), albeit, it still keeps higher blood glucose level (210.1 mg/dl) than sham controls (149.3 mg/ml). In addition, valsartan treatment significantly reduced the plasma TF activity from 11.0 pM to 9.2 pM in STZ-diabetic mice. However, valsartan alone did not significantly influence these parameters compared to sham controls. Of particular interest, expressions of Egr-1, TLR-4 and TF significantly increased in thoracic aorta of STZ-treated animals, however, treatment with valsartan (20 mg/kg) for 8 weeks significantly reduced them in STZ-induced diabetic mice (Fig.8). Administration of valsartan (20 mg/kg) in STZ-induced diabetic mice significantly improved the glucose tolerance (Fig.9).

**Table 1:** Changes of different parameters of mice after 8 weeks of treatment of vehicle, STZ, STZ+valsartan (10 or 20 mg/kg), and valsartan (20 mg/kg)

Treatment	Number of animals	Body weight (g)	Plasma glucose (mg/dl)	Plasma insulin (ng/ml)	Plasma tissue factor activity (pM)
Vehicle	7	29.6 ± 2.4	149.3 ± 13.9	0.88 ± 0.24	7.3 ± 0.9
STZ	10	23.8 ± 1.2**	336.3 ± 54.5**	0.13 ± 0.02**	11.0 ± 1.4**
STZ+Valsartan (10 mg/kg)	10	23.9 ± 1.2	298.2 ± 88.1	0.17 ± 0.10	11.1 ± 2.5
STZ+Valsartan (20 mg/kg)	10	24.2 ± 1.2	210.1 ± 61.4††	0.49 ± 0.10††	9.2 ± 1.6†
Valsartan (20 mg/kg)	7	27.7 ± 1.2	145.4 ± 35.7	0.68 ± 0.13	9.0 ± 1.1

Value are means ± SEM.

**P < 0.01 *versus* Vehicle.

†*P* < 0.05,

††*P* < 0.01 *versus* STZ, respectively.

## Discussion

Recently, it has been suggested that the renin–angiotensin system (RAS) may play a significant role in regulating the AMPK system 19. ARBs are considered to play a protective role in patients with hypertension, heart disease and DM in addition to their anti-hypertensive effect 20,21. Sometimes the effect of ARBs is independent of AT_1_R blockade. For example, telmisartan, acts in skeletal muscle through the stimulation of the PPAR/AMPK pathway 22. The current study demonstrated that valsartan significantly inhibited Egr-1 at transcriptional level which led to inhibition of HG-activated inflammatory cytokine, TLR-2 and-4, and TF expression/activity *via* AMPK activation in THP-1 cells. Egr-1 is highly expressed in human atherosclerotic lesions and is induced in the aorta of LDL receptor-deficient mice following cholesterol feeding 23. Activation of PKC was known in human monocytes to induce TLR by HG 24. In agreement with this, we obtained that pre-treatment with Gö9676, a pan-PKC inhibitor, resulted in reduction of expression of HG-induced Egr-1. Furthermore, we found that among MAPKs, ERK1/2 is responsible for induction of Egr-1. Thus, activation of PKC/ERK1/2 seems to be important signals in Egr-1 regulation in HG condition. Although these two signals play a crucial role for induction of Egr-1 expression in HG condition of THP-1 cells, whether PKC influences ERK activity or ERK activates PKC is not known. Thus, the levels of p-PKC and p-ERK1/2 by HG were examined by corresponding signal inhibitors (Gö6976 or PD98059), respectively. Phosphorylation of PKC by HG was not diminished by PD98059 (ERK inhibitor), but that of ERK1/2 by HG was significantly reduced by Gö6976 (PKC inhibitor), which means PKC is located upstream of ERK1/2 MAPK. Interestingly, either PKC or ERK1/2 pathway is not but AMPK signal involved in inhibition of Egr-1 expression by valsartan. We found that compound C and DN-AMPK-transfected cells exhibited significantly reversed the effect of valsartan. Egr-1 has been reported to induce foam cells and expression of TF by TLR in macrophage cells 10. Indeed, by acting as a master transcription factor, Egr-1, a zinc finger nuclear protein, regulates a set of genes implicated in the pathogenesis of atherosclerosis, with subsequent thrombosis and restenosis 11,12. Recently Arai *et al*. 25 reported that metformin, an AMPK activator, has been shown to attenuate TF expression through inhibition of the ERK1/2/Egr-1 pathway in human monocytes stimulated with LPS or oxidized low-density lipoprotein (oxLDL). Here, we report that valsartan reduced HG-activated TF expression by inhibition of Egr-1 expression *via* AMPK activation. However, it should be noted that at least ERK1/2 signal pathway is not involved in inhibition of Egr-1 by valsartan. The reason why the current results differ from those of Arai *et al*. 25 cannot be explained. However, it is likely that different stimulant, cell sources and experimental conditions may have caused a different outcome; in the current study, HG was used as a stimulator of TF expression, however, they used LPS or oxLDL.

Several metabolic disorders, *e.g*. obesity, type 2 diabetes and atherosclerosis, trigger immune defence mechanisms and induce chronic inflammation, which in turn aggravates the symptoms of the disease 26. It is not surprising if valsartan activates AMPK, it is natural that this drug can reduce inflammation. We demonstrated that valsartan significantly reduced NF-κB-mediated inflammatory cytokines and TLR-2 and-4 expressions by HG in a compound C-sensitive manner. These findings suggest that application of valsartan can be broadened to metabolic disorders, where the immune and inflammation systems are combined in the pathological processes. These observations suggest that inhibition of NF-κB may also play an important role in valsartan inhibition of HG-induced TF expression in THP-1 cells. To confirm that valsartan binds to NF-κB and Egr-1 in the TF promoter, when the ChIP assay was performed, valsartan inhibited binding of these transcription factors to the TF promoter. To understand whether these two transcription factors interact with each other or work independently, when co-immunoprecipitation experiment was done by Western blot analysis, there did not appear to be any difference in the binding of NF-κB to Egr-1 (Fig. S1). These findings suggest that NF-κB and Egr-1 are therapeutic targets of valsartan for reduction of TF expression in hyperglycaemic condition. Indeed, it has been reported that AMPK inhibits NF-κB signalling and inflammation 27. It should be mentioned that treatment with ARBs increase the levels of angiotensin (1-7) and this RAS mediators could take part in AT_1_R independent action of valsartan. ARBs activate ACE2/Ang-(1-7)/Mas axis which is relevant for the anti-inflammatory effects of the ARBs in a rat model of autoimmune myocarditis 28. Thus, it remains to be clarified whether anti-inflammatory action demonstrated by valsartan is partly through the ACE2/Ang-(1-7)/Mas axis. However, this possibility is low. Because (1) HG did not produce Ang II in THP-1 cells and (2) Ang II is the major substrate for Ang-(1-7) synthesis 29,30. We thus propose that effect of valsartan is not associated with AT_1_R blocking action, but activation of AMPK. We found that AMPK activation by valsartan was also demonstrated even in CHO cells, which were devoid of AT_1_R 31. Further supporting evidence comes from the result that valsartan significantly reduced expression of Egr-1, TF, TLR-2 and-4 even in si-AT_1_R-RNA-transfected cells. Furthermore, HG did not increase Ang II concentration in the media of THP-1 cells. Thus, decreased expression of TF, TLR-2 and-4 by valsartan is not related to blockade of AT_1_R in THP-1 cells. Some of pharmacological effect of valsartan which was independent of AT_1_R has been reported 32. We propose that valsartan directly activates AMPK by activating LKB1. Finally, treatment with valsartan resulted in significantly reduced expression of Egr-1, TF and TLR-4 in blood vessel in STZ-induced diabetic mice. We found that valsartan significantly ameliorated glucose tolerance and insulin resistance in STZ-diabetic mice. This finding supports earlier report that ARBs ameliorate glucose uptake *via* the AMPK pathway, which is independent of the insulin pathway 33. It should be noted that AT_1_R blockade is well-established in many articles to lower glucose levels during glucose challenge tests.

In summary and conclusion, we report here for the first time that valsartan reduced HG-induced Egr-1 mRNA and protein expression by the AMPK-dependent pathway, but not PKC/ERK1/2 MAPK pathway. In addition, valsartan can reduce HG-induced TF and TLR-2 and-4 expressions *via* Egr-1 regulation. Valsartan increases expression of p-AMPK by activation of LKB1. The increase in inflammatory cytokines (TNF-α, IL-6 and IL-1β), TLR-4, TF expression and activity by HG were significantly decreased by valsartan because of inhibition of NF-κB activity (Fig.10). In STZ-induced type 1 diabetic mice, treatment with valsartan resulted in significantly reduced Egr-1, TF and TLR-4 in aorta. In addition, valsartan induced significant improvement of glucose tolerance and reduced insulin levels in STZ mice. However, it should be noted that although valsartan treatment statistically improved glucose homoeostasis, those parameters such as blood glucose and insulin levels are still higher than sham controls. Thus, this effect is specific only to valsaratan or other class of ARBs. Taken together, it is concluded that valsartan inhibits TLR-2,-4 expression, TF expression and activity in hyperglycaemic conditions *via* activation of AMPK, which directly inhibits Egr-1 expression (Fig.10) in human macrophage cells and in aorta of STZ-induced diabetic mice.

**Figure:**
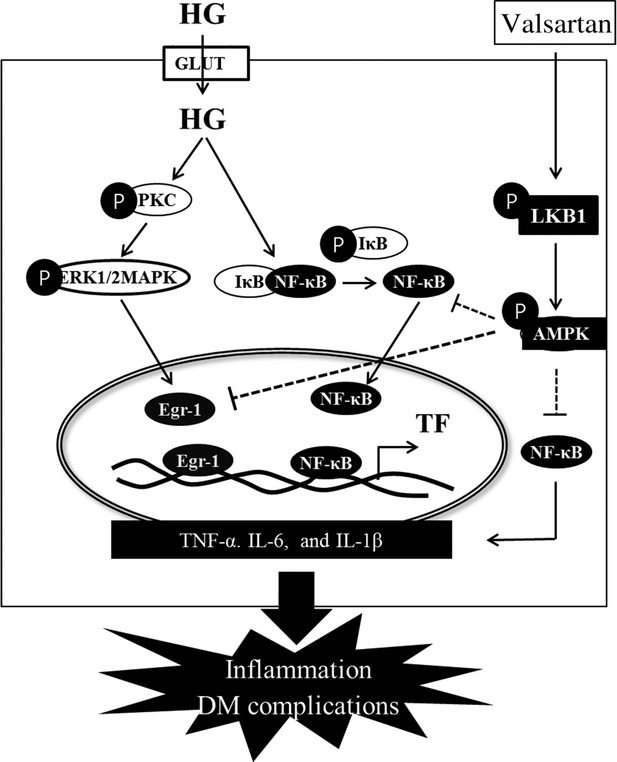
Schematic signalling pathway of valsartan to inhibit tissue factor (TF) expression and inflammatory cytokines in high glucose (HG) condition. High glucose (HG) can be uptaken inside the cell, where it activates NF-κB and protein kinase C (PKC) by phosphorylation. The activated PKC then stimulates phosphorylation of ERK1/2 MAPK which regulates transcription of Egr-1. On the other hand, the activated NF-κB moves into nucleus and induces pro-inflammatory cytokines such as TNF-α, IL-6 IL-1β *etc*. In the nucleus, Egr-1 and NF-κB bind to TF promoter and induces TF expression. Valsartan induces phosphorylation of AMPK *via*LKB1 activation. The activated AMPK directly inhibits Egr-1 transcription, independent of PKC/ERK signal, so that TF expression is inhibited in HG condition. In addition, valsartan inhibits NF-κB translocation in HG condition, which reduces production of NF-κB-based pro-inflammatory cytokines. Thus, administration of valsartan, independent of AT_1_R, may inhibit pro-inflammatory cytokines and TF expression in diabetic conditions *in vitro* and *in vivo*, thus it reduces inflammation and other complications associated with type 1 diabetes mellitus.

## Supporting Information

Figure S1 Co-immunoprecipitation (IP) assay and Western blot analysis.
