# Supplementary figures and images for: Valsartan independent of AT1 receptor inhibits tissue factor, TLR-2 and-4 expression by regulation of Egr-1 through activation of AMPK in diabetic conditions
Source: J Cell Mol Med. 2014 Aug 11;18(10):2031–43. doi: 10.1111/jcmm.12354 (PMC4244018; doi:10.1111/jcmm.12354)

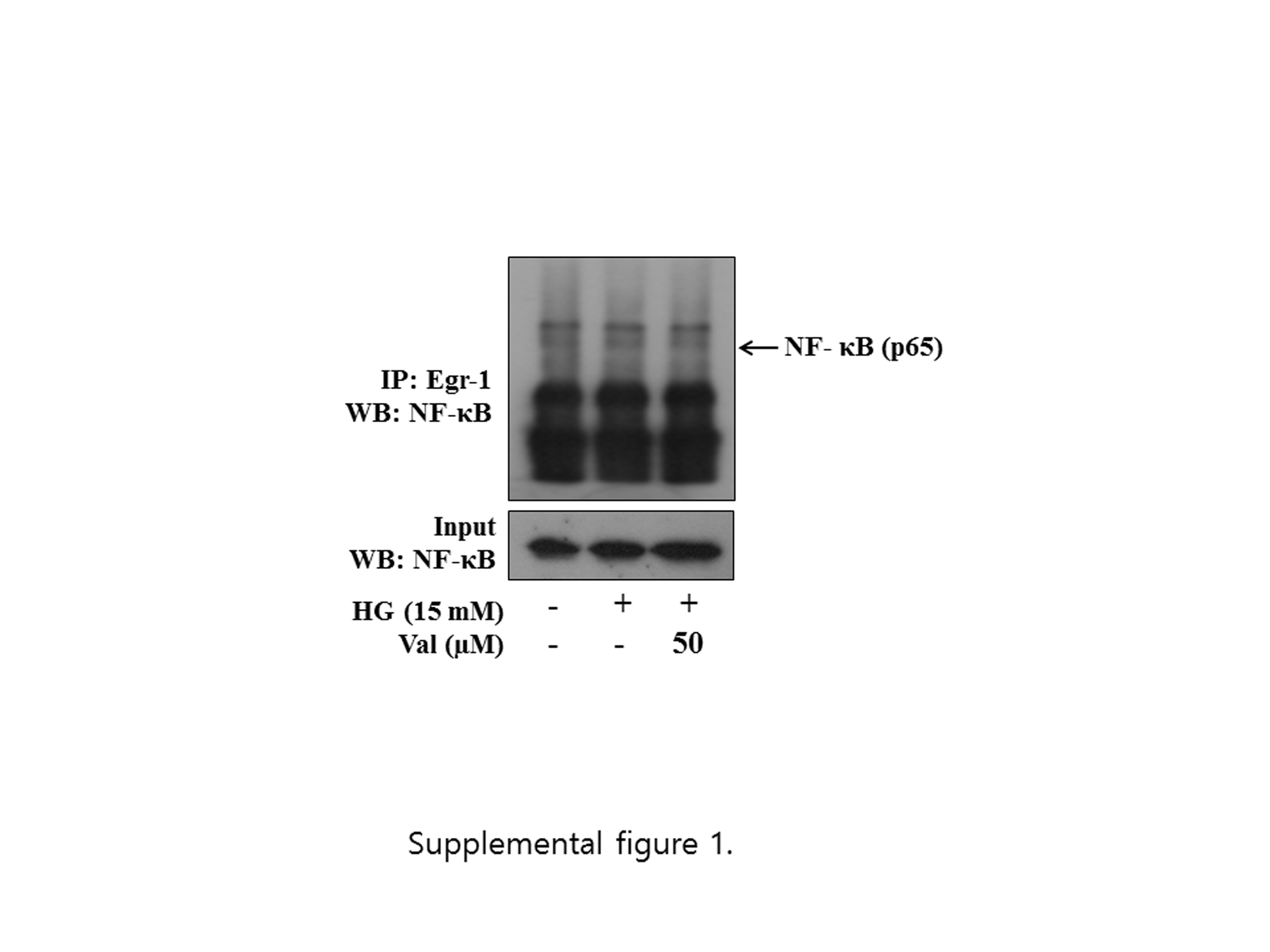

Supplement: Figure S1 — Co-immunoprecipitation (IP) assay and Western blot analysis. [file jcmm0018-2031-sd1.tif]
