# Supplementary material for: Valsartan independent of AT1 receptor inhibits tissue factor, TLR-2 and-4 expression by regulation of Egr-1 through activation of AMPK in diabetic conditions
Source: J Cell Mol Med. 2014 Aug 11;18(10):2031–43. doi: 10.1111/jcmm.12354 (PMC4244018; doi:10.1111/jcmm.12354)
Supplement: Supplementary file 2 [file jcmm0018-2031-sd2.docx]

**Supplement**

**Valsartan independent of AT_1_ receptor inhibits tissue factor, TLR-2 and -4 expression by regulation of Egr-1 through activation of AMPK in diabetic conditions**

**Methods**

**Co-immunoprecipitation and western blotting**

To know whether NF-κB and Egr-1 interact with each other or work independently, co-immunoprecipitation experiment was performed. THP-1 cells were plated at a density of 2.5 × 10^6^ cells per ml in a 100 mm dish. To induce macrophage phenotype differentiation, 50 ng/ml phorbol 12-myristate 13-acetate was added to the culture. After 24 h, non-adherent cells and PMA were washed off three times with phosphate buffered saline. Then cells were treated with valsartan 1 h before stimulation with HG (15 mM). After 1 h incubation, whole cell lysates extracted in lysis buffer with protease inhibitor cocktails. Protein concentration of the cell lysates was determined using a Bradford assay. For immunoprecipitation, 100 µg of the cell protein was incubated with anti-Egr-1 antibody overnight at 4℃ on a rotator. Immunocomplexes were added with protein A/G PLUS-Agarose beads for 3 h. Beads were washed 3 times with lysis buffer, and protein complexes were eluted by adding sample buffer. Immunoprecipitated proteins were determined by western blot analysis using the primary anti-NF-κB antibody (1:1000).

**Results**

As shown in supplemental Fig.1, there did not appear to be any difference in the binding of NF-kB to Egr-1 in the presence or absence of valsartan (50 μM) in high glucose (15 mM)-treated THP-1 cells. This result indicates that NF-kB and Egr-1 interact independently to express TF protein in HG-activated THP-1 cells.

**Figure legend**

Co-immunoprecipitation (IP) assay and western blot analysis. Cell lysates were subjected to a Co-IP assay with anti- Egr-1-antibody and were immunoblotted (WB) with anti-NF-kB-antibody.
